# High Efficacy of Preoperative Low-Dose Radiotherapy with Sanazole (AK-2123) for Extraskeletal Ewing's Sarcoma: A Case Report

**DOI:** 10.1155/2011/185465

**Published:** 2010-09-06

**Authors:** Tomoya Sakabe, Hiroaki Murata, Eiichi Konishi, Kazutaka Koto, Naoyuki Horie, Takaaki Matsui, Yasushi Sawai, Hideya Yamazaki, Tsutomu V. Kagiya, Toshikazu Kubo

**Affiliations:** ^1^Department of Orthopedics, Graduate School of Medical Science, Kyoto Prefectural University of Medicine, 465 Kawaramachi-Hirokoji, Kamigyo-ku, Kyoto 602-8566, Japan; ^2^Division of Surgical Pathology, Graduate School of Medical Science, Kyoto Prefectural University of Medicine, 465 Kawaramachi-Hirokoji, Kamigyo-ku, Kyoto 602-8566, Japan; ^3^Department of Radiology, Graduate School of Medical Science, Kyoto Prefectural University of Medicine, 465 Kawaramachi-Hirokoji, Kamigyo-ku, Kyoto 602-8566, Japan; ^4^Health Research Foundation, Kinki Invention Center, 14 Yoshida-Kawaramachi, Sakyo-ku, Kyoto 606-8255, Japan

## Abstract

Extraskeletal Ewing's sarcoma is a rare soft tissue tumor that is morphologically indistinguishable from Ewing's sarcoma of bone. We report a case of extraskeletal Ewing's sarcoma with several systemic problems. A 69-year-old man presented with a 5-month history of a rapidly enlarging mass in the right thigh. Because preoperative radiotherapy with sanazole (AK-2123) contributed the tumor mass reduction down to 40% in size, the tumor was successfully resected with clear surgical margins and repaired with a musculocutaneous flap. The high efficacy of pre-operative low-dose radiotherapy with sanazole was histologically confirmed that the resected tumor specimen involved no viable tumor cells and showed 100% necrosis. Based on clinical outcomes in this case, the combined modality of pre-operative low-dose radiotherapy with hypoxic cell radiosensitizer and adequate surgical resection might provide for the useful clinical application of extraskeletal Ewing's sarcoma treatment.

## 1. Introduction

Extraskeletal Ewing's sarcoma (EES) is a rare type of Ewing's sarcoma that arises in the soft tissue at any location and is now regarded as a member of a family of small cell neoplasms of bone and soft tissue, including primitive neuroectodermal tumors (PNETs) [1, 2].Multimodal treatment combined with surgical treatment, chemotherapy, and radiotherapy (RT) contributes the improvements of clinical outcomes in EES [3]. Definitive local control of Ewing's sarcoma is a prerequisite of cure and local failures are associated with an extremely poor prognosis [4]. Although RT has been the local treatment of choice in Ewing's sarcoma, the clinical impact on survival by RT is controversial [3, 5]. Postoperative RT is mainly given in case of inadequate surgical margins or poor response to chemotherapy [4]. However, the benefits, indication, and optimal timing of adjuvant RT (pre-operative versus post-operative) remain unclear [6]. As compared with post-operative RT, the relative advantages of pre-operative RT include smaller and well-defined treatment volume, ability to use a lower dose, lack of tissue hypoxia, increased tumor resectability, and improved limb function with less late fibrosis and edema [6]. 

Efficacy of RT largely depends on tumor radiosensitivity and the tolerance of normal tissues. To improve therapeutic outcomes, RT is often combined with chemotherapeutic drugs that are themselves cytotoxic and/or radiosensitizing. Sanazole (AK-2123) as a radiosensitizer with irradiation in various tumors showed encouraging results [7–9]. Here, we describe a case of EES that were successfully treated with surgical resection after pre-operative low-dose RT and additional administration of sanazole as a hypoxic cell radiosensitizer.

## 2. Case Report

**Figure 1:**
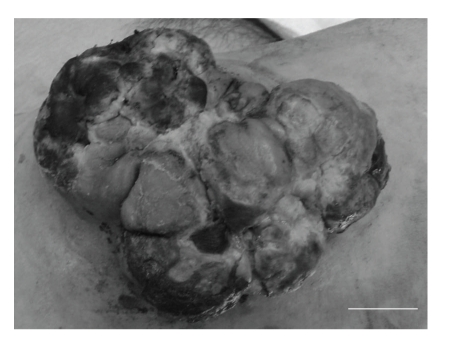
Macroscopic findings of the tumor in the pre-treatment condition. The tumor involved multiple ulcers and necrotic tissues. Bar = 2 cm.

**Figure 2:**
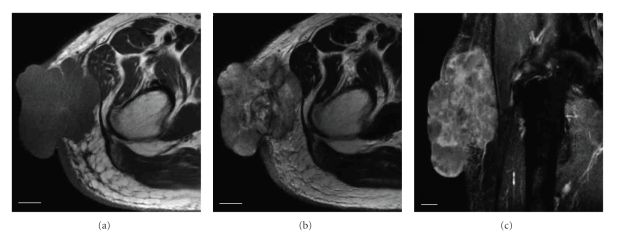
MR images of the tumor in the pre-treatment condition. (a) Axial T1-weighted image. (b) Axial T2-weighted image. (c) Coronal STIR sequence enhanced with Gd-DTPA. Bars = 2 cm. The heterogeneous mass was attached to the fascia lata and expanded across the skin layer.

**Figure 3:**
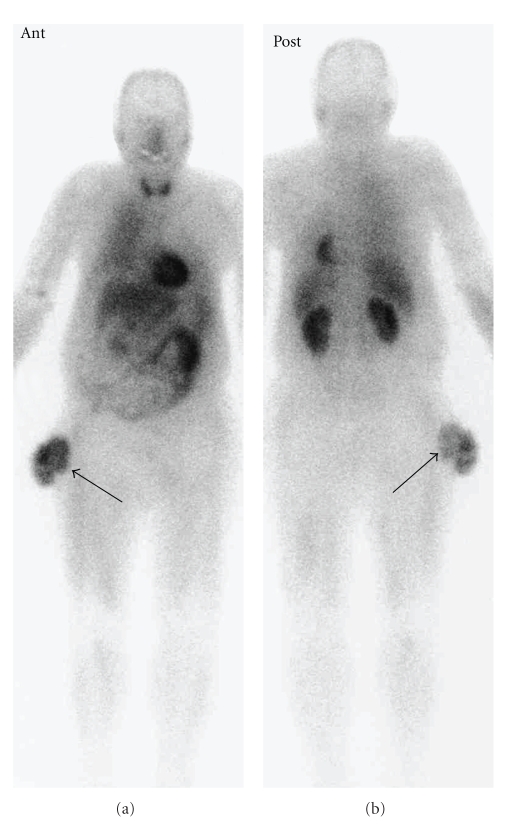
Thallium-201 scintigram of whole body in early phase. Abnormal accumulations accorded for the mass were detected (black arrows).

A 69-year-old man presented at our hospital with a 5-month history of a rapidly enlarging mass in the right thigh. His past history was pulmonary tuberculosis treated with thoracoplasty and hepatitis C. Physical examination revealed the presence of a large irregular mass with multiple ulcers and necrotic tissues, measuring approximately 10 × 10 cm in the lateral side of the proximal thigh (Figure 1). Admission laboratory data showed high levels of LDH, GOT, GPT, and ALP, which are 643, 80, 39, and 441, respectively. Typical tumor markers were within normal limits. A roentgenogram of the right proximal thigh showed no apparent expansion to the femur. Chest roentgenogram and computed tomography showed no apparent metastatic lesion and no focus of pulmonary tuberculosis. MR images demonstrated relatively a well defined and heterogeneous mass, which was attached to the fascia lata and expanded across the skin layer (Figures 2(a), 2(b)). The signal intensity of the mass was isointense to the adjacent skeletal muscles on T1-weighted images and inhomogeneous hyperintense on T2-weighted images. The mass was relatively well defined and enhanced with Gd-DTPA on STIR sequences (Figure 2(c)). However, partial infiltrating expansions to subcutaneous tissues of the mass made its margins unclear. Despite the mass contacted with the compression to the tensor fascia lata muscle, the gluteus maximus muscle, and the gluteus medius muscle, no apparent signal intensity changes of muscles were seen. Thallium-201 scintigrams showed a robust accumulation accorded for the mass in both of early and delayed phases (Figure 3). No other abnormal accumulation was detected.

**Figure 4:**
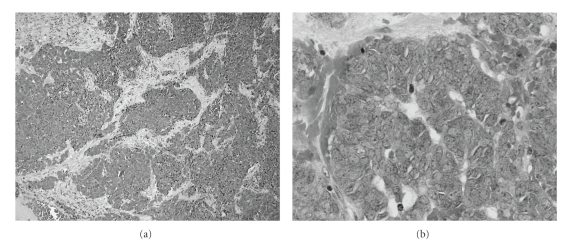
Histological findings of the HE-stained biopsy sections. (a) Low power view (magnification: x6). Cellular nodules are scatters within fibrous stroma. (b) High power view (magnification: x36). The tumor cells have oval nuclei with scant cytoplasms. Mitotic figures are common.

Based on clinical findings and imaging characteristics, the tumor was diagnosed as a primary soft tissue tumor and an incisional biopsy was performed. Histopathology of the permanent sections showed the nodular or sheets-like proliferation pattern of small round cells with a high nuclear/cytoplasmic ratio in the background of sparse extracellular collagen (Figure 4). Immunohistochemistry of the sections demonstrated that the staining of BCL2, MIC2, CD56, O-13, neuron specific enolase (NSE), and synaptophysin were positive. They were negative for HMB-45, S-100, broad cytokeratin (AE1/AE3 and MNF116), CD45, CD79a, CD3, and CD20. In addition, the RT-PCR showed the presence of EWS/FLI-1 fusion transcript from tumor specimens. Together with the results of several examinations, we diagnosed this tumor as EES.

**Figure 5:**
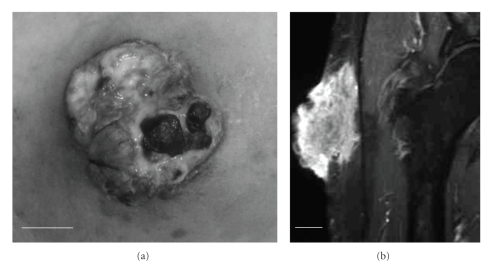
The tumor mass reduction induced by pre-operative RT. (a) Macroscopic findings of the tumor. (b) Coronal STIR sequence enhanced with Gd-DTPA. Bars = 2 cm. The size of the tumor reduced to approximate 40% of the pre-treatment condition.

**Figure 6:**
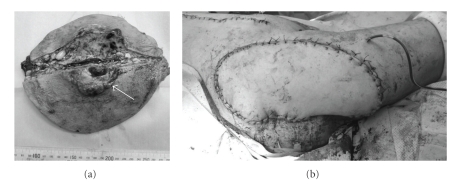
(a) Macroscopic findings of the resected tumor with adjacent soft tissues. Note that the specimen was longitudinally dissected across the tumor (white arrow). (b) Post-operative findings repaired with a musculocutaneous flap of rectus abdominis.

**Figure 7:**
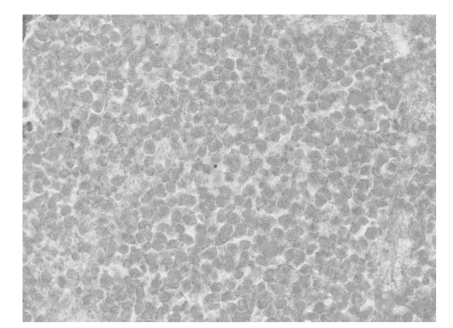
Histological findings of the HE-stained resected sections (magnification: x36). The tumor cells completely showed necrotic appearances.

Considering patient's age, hepatic dysfunction, and patient's disagreement with chemotherapy, we precluded the choice of the neoadjuvant chemotherapy. To define surgical margins especially at subcutaneous tissues and to increase tumor resectability, we determined to treat the patient with pre-operative low-dose RT and additional administration of sanazole before surgery [10]. As a result of the total dose irradiation of 30 Gy in 15 fractions with sanazole (0.6 g/m^2^  × 10 days), the tumor volume was reduced to approximately 40% of the pre-treatment condition (Figure 5). On thallium-201 scintigrams, the accumulation of the tumor markedly decreased in both of early and delayed phases. Subsequently, we could perform the surgical resection with subtotal resection of the tensor fascia lata muscle, the gluteus maximus muscle, the gluteus medius muscle, and adjacent soft tissues to obtain wide surgical margins (Figure 6(a)). The huge surgical defect in the lateral thigh was repaired with a musculocutaneous flap of rectus abdominis (Figure 6(b)). There was no major complication in the perioperative period. Histological examination of the resected tissue demonstrated clear surgical margins. Because the tumor cells completely showed necrotic appearances and there were no viable tumor cells in the tumor specimen, we diagnosed the necrosis ratio as 100% (Figure 7). Based on the histological findings, we could confirm a high efficacy of pre-operative low-dose RT with sanazole in the present case. The patient underwent follow-up without any local recurrence or distant metastasis, and presented without any postoperative functional disturbance for 24 months after surgery.

## 3. Discussion

EES is a tumor of unknown mesenchymal origin predominantly affecting men in the second and third decades of life [2, 11]. The most frequent sites of occurrence are the chest wall, lower extremities, and paravertebral region [12]. Less frequently, the tumor occurs in the pelvis and hip region, the retroperitoneum, and the upper extremities [12]. Although EES is rare and very few data are available addressing optimal surgical and oncologic modalities, EES should be considered in the differential diagnosis of soft tissue tumor including embryonal rhabdomyosarcoma, lymphoma, and neuroblastoma [1, 13]. EES and Ewing's sarcoma of bone have similarities for natural history, histological findings, immunohistochemical staining results, and molecular biological characteristics [5, 13].The patients with EES, like its osseous counterpart, should be treated with the multimodal treatment including appropriate surgery, multiagent chemotherapy and high-dose RT [13]. Raney et al. reported a 10-year survival rate between 61%–77% utilizing multiagent chemotherapy [14]. However, treatment outcome after chemotherapy and RT has not yet been determined [1, 5, 13–16]. Therefore, complete removal of the tumor is essential whenever feasible. Complete resection with wide surgical margins should remain the goal of surgery because those patients who undergo a wide resection can improve overall survival compared with those patients treated with inadequate surgical margins or without surgical resection [5]. 

Despite the role of RT in the management of bone sarcoma is limited, its primary application appears to be in Ewing's sarcoma [6]. The importance of systemic treatment by chemotherapy and its histological responses as a prognostic factor for outcome has been recognized for some time [3, 4]. However, several large series demonstrate that improvement in local control has been the major route to improved outcomes in Ewing's sarcoma including EEF [3]. In the patients with unfavorable locations (e.g., spine, pelvis) or large residual tumors after induction chemotherapy, the risk of local failures is high after surgery alone because adequate resection is difficult to achieve [3, 17]. The addition of post-operative RT in these patients has resulted in improved rates of local control and survival outcomes [3]. Current consensus favors the use of post-operative RT in all patients with inadequate surgical margins and/or poor responder to chemotherapy [3, 6]. On the other hand, there is no difference between pre-operative RT and post-operative RT in local control, distant control, or survival rates despite the incidence of wound complications is higher in pre-operative RT than in post-operative RT [6]. When a negative surgical margin is obtained following pre-operative RT, local failure rates are comparable with those achieved with negative margin surgery [17]. Therefore, pre-operative RT could be an attractive application to avoid inadequate surgical margins in case of large tumors or tumors in unfavorable locations. In addition, RT with hypoxic cell radiosensitizer is often used to improve therapeutic outcomes [18]. Sanazole (AK-2123) was presented as a new type of hypotoxic cell radiosensitizer owing to its low-toxicity (e.g., reversible peripheral neuropathy) and high-radiosensitizing effects and has been tested clinically in various tumors [7, 18]. Following these reports, the present case with large EES underwent pre-operative RT with 30 Gy and additional administration of sanazole to increase the tumor resectability. Despite the total dose of radiation in post-operative RT depends on the extent of resection, the relevant dose of pre-operative RT avoiding complications remains unclear. The recommended radiation doses are 55.8 Gy for gross residual disease and 50.4 Gy for microscopic disease after surgical resection [19]. Considering the higher risk of acute wound healing complications in pre-operative RT, we determined to deliver the total dose of 30 Gy in 15 fractions which is relatively low dose [16, 20, 21]. Consequently, we could achieve the tumor size reduction to 40% of the pre-treatment condition and obtain the clear surgical margin without any complications. The best threshold for the extent necrosis for the addition of post-operative RT is yet unknown. However, the local control rates with more than 90% necrosis seem low enough for omitting RT [3]. From the microscopic results of the resected specimen with 100% necrosis, we omitted the addition of post-operative RT in the present case. 

Despite the limitation of this case including its short follow-up period, our clinical outcomes in radiosensitive EES suggested the high efficacy of pre-operative low-dose RT with sanazole. Therefore, the combined modality of pre-operative low-dose RT with hypoxic cell radiosensitizer and adequate surgical resection might provide for the useful clinical application of EES treatment.
